# Surgical Management of Tentorial Notch Meningioma Guided by Further Classification: A Consecutive Study of 53 Clinical Cases

**DOI:** 10.3389/fonc.2020.609056

**Published:** 2021-01-22

**Authors:** Chaoying Qin, Junquan Wang, Wenyong Long, Kai Xiao, Changwu Wu, Jian Yuan, Yimin Pan, Chi Zhang, Jun Su, Xianrui Yuan, Qing Liu

**Affiliations:** ^1^Department of Neurosurgery in Xiangya Hospital, Central South University, Changsha, China; ^2^Institute of Skull Base Surgery & Neuro-oncology at Hunan Neurosurgery Institute of Central South University, Changsha, China

**Keywords:** tentorial notch meningioma, further classification, microsurgery, surgical approach, neurofunction protection

## Abstract

**Background:**

Management of tentorial notch meningiomas (TNM) remains a challenge for neurosurgeons. We demonstrate the clinical characteristics and surgical experiences of TNM based on our cases according to a proposed further classification.

**Methods:**

We retrospectively analyzed clinical and follow-up data in a consecutive series of 53 TNM patients who underwent microsurgical operation from 2011 to 2019 in our institution. The operations were performed using various approaches. Clinical history, preoperative and postoperative neurofunction, imaging results, and surgical outcomes were collected for further classification of TNM.

**Results:**

All TNM cases were divided into anterior (T1), middle (T2), and posterior notch (T3). According to the direction of tumor extension and correlation with the neurovascular structures, detailed subtypes of anterior TNMs were identified as the central (T1a), posterior (T1b), and medial type (T1c). The middle TNMs were divided into the infratentorial (T2a), supratentorial (T2b), and supra-infratentorial type (T2c). The posterior TNMs were divided into superior (T3a), inferior (T3b), lateral (T3c), and straight sinus type (T3d) in reference to Bassiouni’s classification. Total removal of the tumor was achieved in 46 cases, with five cases of subtotal and two cases of partial removal without any recorded deaths in our series. In total, five subtotal resected cases underwent gamma-knife treatment and achieved stable disease. Postoperative aggravation or new onset cranial nerve dysfunction occurred in some individual cases, with incidences ranging from 3.77 to 15.10% and improved preoperative neurological deficits ranging from 0 to 100%.

**Conclusion:**

Further, TNM classification based on the intracranial location, extension direction, relationship with brainstem, and neurovascular structures guides preoperative evaluation, rational surgical approach selection, and surgical strategy formulation. Taking microsurgery as the main body, a satisfactory outcome of TNM treatment can be achieved for complicated tumors by combining stereotactic radiotherapy. This study demonstrates the surgical outcomes and complications in detail. Further classification might be helpful for treatment decisions in the future.

## Introduction

Tentorial meningioma was first reported by Andraal in 1833 (1). Nowadays, they are often defined as a type of posterior fossa meningioma, accounting for about 3% of intracranial meningiomas (2). In the early stage, surgical intervention was limited due to a lack of preoperative imaging information and poor prognostic techniques. With the continuous progress in microsurgery and the advent of imaging developments, the prognosis of meningiomas has improved significantly. However, tentorial meningiomas, especially those located at the free margin of the tentorium, which are named after tentorial notch meningioma (TNM), are still challenging for neurosurgeons.

Common clinical symptoms of TNM are variable, including headache, dizziness, hearing loss, tinnitus, facial numbness, facial pain, ataxia, mild hemiplegia, diplopia, vision loss, visual field defect, difficulty in swallowing, choking, etc. (3–5). Yaşargil divides TNM into three types: anterior, middle, and posterior (T1, T2, T3) according to their different locations corresponding to the three portions of the free margin of the tentorium (6). Although it is risky and difficult, the preferred treatment for TNM is surgical resection.

First, the location of the TNM is deep inside the intracranial space, as the tentorial notch is located at the depth of the midline and para-midline of the skull base, which is close to the midbrain. Specific surgical approaches are needed to reach and expose deep lesions. Resection is inevitably limited by the long anatomical distance and restricted operating space. Specific surgical approaches often contain corresponding risks.

Second, the tumor invades the cranial nerves, main arteriovenous structures, and the brainstem. Meningiomas located at the anterior notch mainly invade the oculomotor nerves (CN III), abducens nerves (CN VI), anterior choroidal artery (AChA), posterior communicating artery (PComA), and posterior cerebral artery (PCA) and are able to compress the brainstem posteriorly and medially and indirectly compress the diencephalon. Meningiomas located at the middle notch mainly affect the trochlear nerve (CN IV), trigeminal nerve (CN V), facial-auditory nerve (CN VII–VIII), AChA, P2 segment of the PCA, superior cerebellar artery (SCA), and basal vein. Those with larger volumes may compress the brainstem, causing a contralateral shift. The posterior notch space is mainly composed of a complicated deep venous system. Tumors may compress the cranial nerves, even deforming or enveloping them. The arterial structure can be clamped and wrapped, and the functional small penetrating arteries may be enveloped by the tumor, especially the branches of the anterior perforated substance and the brainstem surface (7). Meningiomas of the posterior notch often invade the venous sinuses. Removing the tumors may damage the adjacent veins, resulting in obstruction of the deep vein system (8). At present, preservation of neurovascular function is increasingly valued by neurosurgeons (7, 9).

Tumors can extend bilaterally along the cerebellar tentorium and invade the dura mater at the skull base. It is always variable in the anterior notch space. They can extend along the lateral wall of the cavernous sinus (CS) to the middle cranial fossa, directly invade the oculomotor triangle into the cavernous sinus, and extend to the midline along the dorsum sellae and posterior clinoid process. The middle TNMs are located at the infra, supra, and supra-infratentorium and further elongate the middle cranial fossa and dorsal petrosum. The posterior type is the so-called falcotentorial junction meningioma (3, 10). The extent of the tumor closely correlates with the surgical strategy.

It is difficult to achieve tumor eradication when the lesion reaches a large volume, and meningiomas are often rich in blood supply. Intraoperative bleeding and diminishing the operating space both increase the difficulty of visualizing and protecting vital structures. Therefore, early exposure and management of the blood supply and the order of tumor debulking are critical for crafting the surgical strategy.

In addition, with regard to the classification of TNM, the tumors are mostly divided by the anterior, middle, and posterior parts of the free margin of the notch (3, 11), which is too simple to sufficiently distinguish TNM and the other meningiomas of parasellar and petroclival regions, such as cavernous sinus meningioma, anterior clinoid process meningioma, and true petroclival meningioma addressed by Al-Mefty (12). Overlap of the identification can be induced, which is not conducive to further detailed treatment.

In this study, we retrospectively analyzed the perioperative and follow-up data of 53 cases of TNM admitted in the past 8 years by our institution. Further classification of TNM is proposed, and the selection of surgical strategies and approaches is discussed to explore individualized treatment of TNM, improve the preoperative symptoms, reduce postoperative complications, and elevate the tumor control rate.

## Materials and Methods

### Patient Population

We retrospectively reviewed the clinical records, neuroimaging, and follow-up data of 53 cases of TNM treated microsurgically from January 2011 to June 2019 at our institutions (Xiangya Hospital, Central South University, China). All surgical procedures were approved by the ethics committee of Xiangya Hospital and the patients’ family members, who provided written consent for publication of this study. The patients and their families are able to share their perspective on the treatment they receive with an average age of 52.52 ± 9.5, a total of 12 males (22.6%) and 41 females (77.4%) with a male to female ratio of 1:3.42. One patient was admitted due to tumor progression after gamma knife treatment, and one due to partial removal in another hospital in our series.

### Clinical Manifestations

The main clinical manifestations included 30 cases of headache and dizziness, 15 cases of hearing loss and tinnitus, 14 cases of facial numbness and facial pain, 13 cases of walking instability, 10 cases of limb weakness, 7 cases of vision loss, 6 cases of facial paralysis and taste loss, 4 cases of dysphagia and choking, 5 cases of nausea and vomiting, 3 cases of unconsciousness, 2 cases of abnormal diplopia, and 2 cases of amnesia were observed in our series.

### Preoperative Neuroimaging

All patients in this study were examined preoperatively by MRI of T1, T2, and T1-contrast-enhanced sequences. The posterior TNM and a portion of the anterior and middle TNM patients were examined by CTV or MRV. CTA was performed for the anterior and middle TNMs that closely adhered to important vessels. No patient underwent angiography.

### Tumor Classification

**Figure 1:**
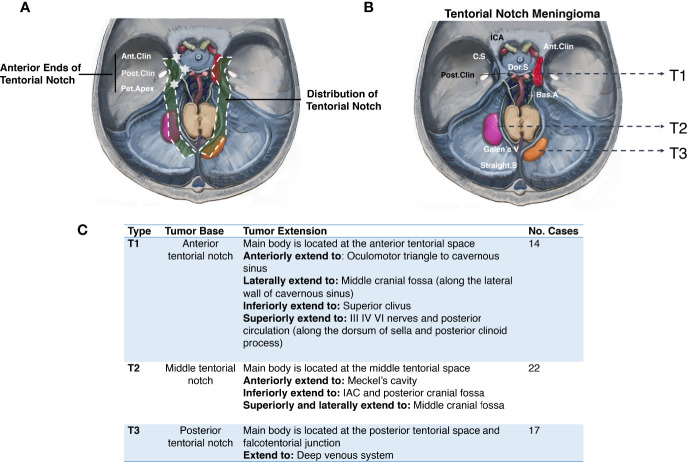
Primary classification of tentorial notch meningioma (TNM). **(A)** The schematic diagram of the distribution of tentorial notch. **(B)** Schematic diagram of the primary classification of tentorial notch meningioma (TNM). T1: Anterior tentorial notch meningioma; T2: Middle tentorial notch meningioma. T3: Posterior tentorial notch meningioma. ICA, Internal carotid artery; Ant.Clin, Anterior clinoid process; C.S, Cavernous sinus; Dor.S, Dorsum Sellae; Bas.A, Basal artery; Galen’s V, Galen’s Vein; Straight S, Straight sinus; Post.Clin, Posterior clinoid process. **(C)** Interpretation of primary classification of tentorial notch meningioma (TNM).

MRI provides the basis for TNM preoperative classification. Based on the falcotentorial meningioma classification proposed by Yaşargil in 1996 (6), we propose the location of the main body and tumor base, and the extension of the base as the classification criteria for distinguishing TNMs from the meningiomas originating from the adjacent dura and to establish a foundation for optimal treatment. TNMs are primarily classified by T1–T3 according to their origins on the distribution of tentorial notches (**Figure 1**).

**Figure 2:**
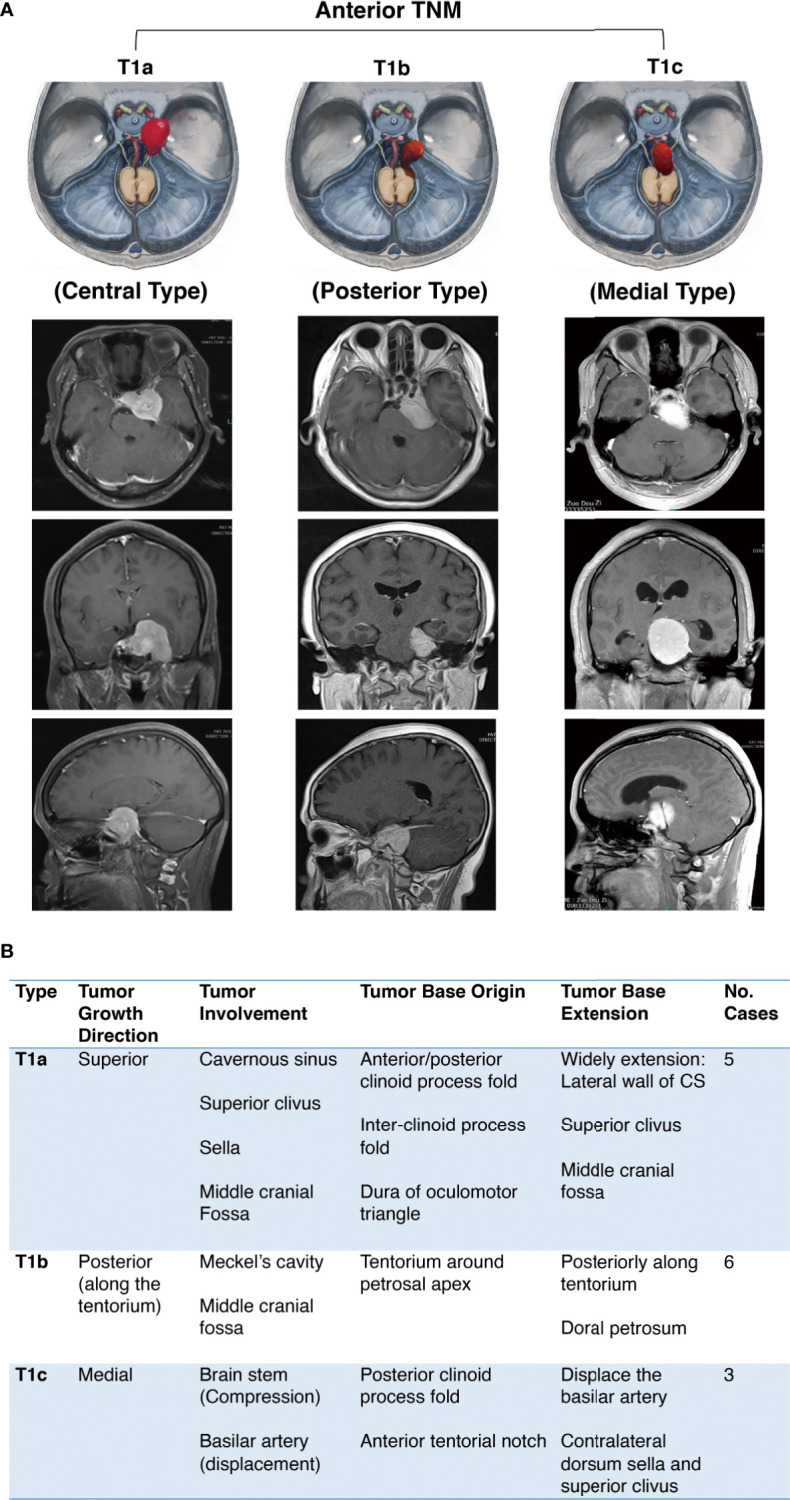
Further classification of anterior tentorial notch meningioma (TNM). **(A)** Schematic diagram and axial, coronal, sagittal MRI T1-weighted images with gadolinium-based contrast of further classification of anterior tentorial notch meningioma (T1). T1a = central type, T1b = inferior type, T1c = medial type. **(B)** Interpretation of further classification of anterior tentorial notch meningioma (TNM).

We divided the anterior TNM into three subtypes according to the growth direction, tumor involvement, and the origin and extension of the tumor base (**Figure 2**):

Subtype T1a: Central type
Subtype T1b: Inferior type
Subtype T1c: Medial type

The inferior and medial types are easily diagnosed as petroclival meningioma through neuroimaging. The identification needs to be confirmed intraoperatively. Notably, the T1b and T1c TNMs originate from the cerebellum tentorium notch, not the dura of the petroclival junction medial to the trigeminal nerve, which is the true origin of petroclival meningiomas (13).

The middle notch is divided into the following subtypes:

Infratentorial **(T2a)**
Supratentorial **(T2b)**
Supra-infratentorial **(T2c)**

**Figure 3:**
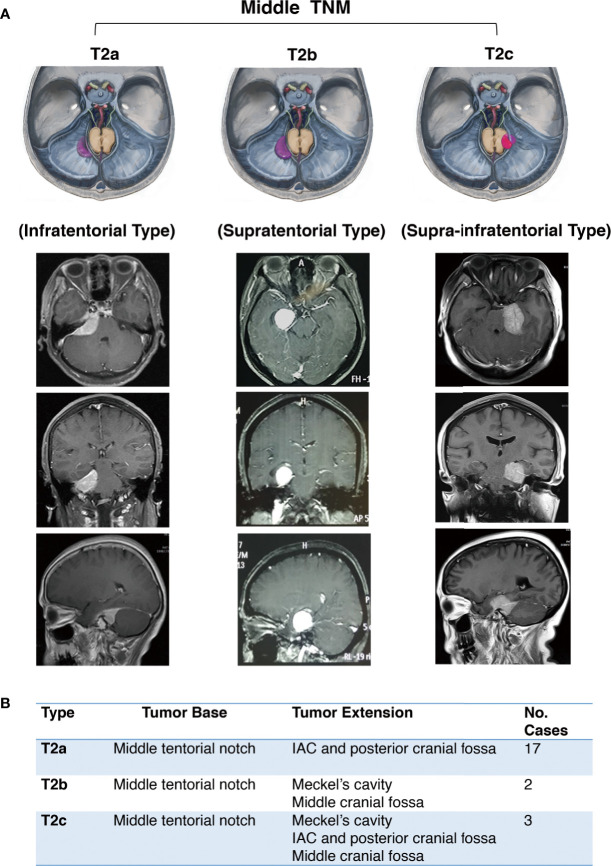
Further classification of middle tentorial notch meningioma (TNM). **(A)** Schematic diagram and axial, coronal, sagittal MRI T1-weighted images with gadolinium-based contrast of further classification of middle tentorial notch meningioma (T2). T2a = infratentorial type, T2b = supratentorial type, T2c = supra-infratentorial type. **(B)** Interpretation of further classification of middle tentorial notch meningioma (TNM).

Anatomically, the main body of T2a TNM is below the tentorium, which can involve part of the dorsal dura of the petrosum. The T2b TNM grows superiorly on the tentorium without infratentorial extension. The T2c refers to tumors growing along both sides of the tentorium, and the middle and posterior skull base dura can both be involved (**Figure 3**).

**Figure 4:**
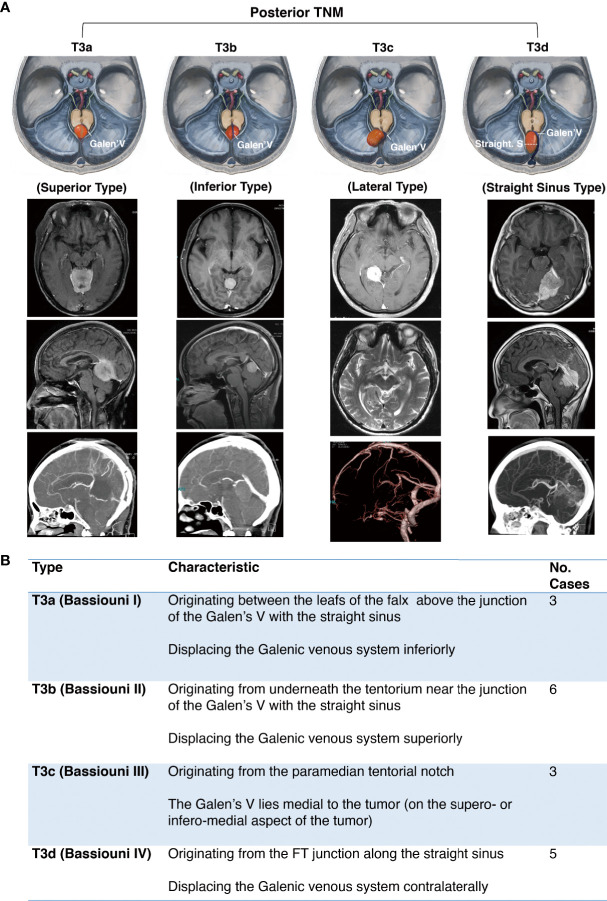
Further classification of posterior tentorial notch meningioma (TNM). **(A)** Schematic diagram and axial, coronal, sagittal MRI T1-weighted images with gadolinium-based contrast of further classification of posterior tentorial notch meningioma (T3). T3a = superior type, T3b = inferior type, T3c = lateral type, T3d = straight sinus type. **(B)** Interpretation of further classification of posterior tentorial notch meningioma (TNM).

The posterior notch TNM was defined in accordance with Bassiouni’s classification in 2008 (10) (**Figure 4**).

### Surgical Methods

**Figure 5:**
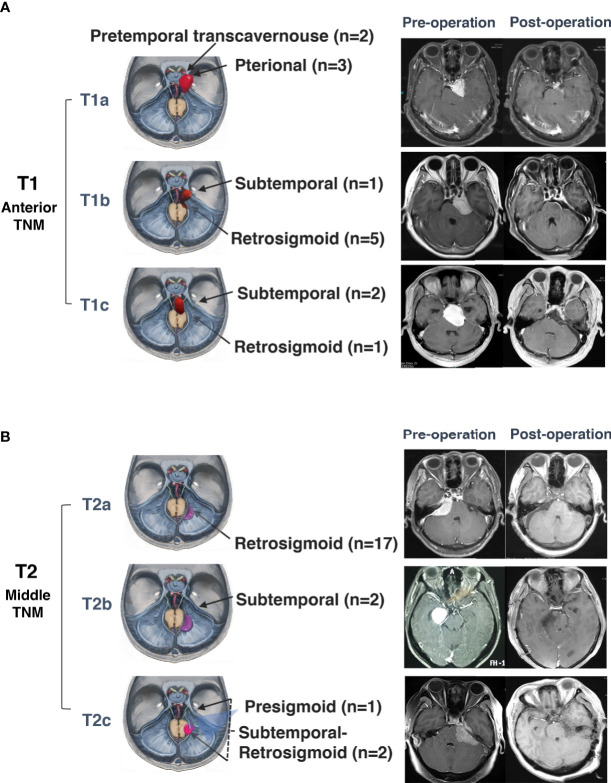
Surgical approaches and outcomes of anterior and middle tentorial notch meningioma (TNM). **(A)** Schematic diagram of surgical approaches and preoperative and postoperative MRI T1-weighted images with gadolinium-based contrast of further classification of anterior tentorial notch meningioma (T1). T1a = central type, T1b = inferior type, T1c = medial type. **(B)** Schematic diagram of surgical approaches for and preoperative and postoperative MRI T1-weighted images with gadolinium-based contrast of further classification of middle tentorial notch meningioma (T2). T2a = infratentorial type, T2b = supratentorial type, T2c = supra-infratentorial type.

**Figure 6:**
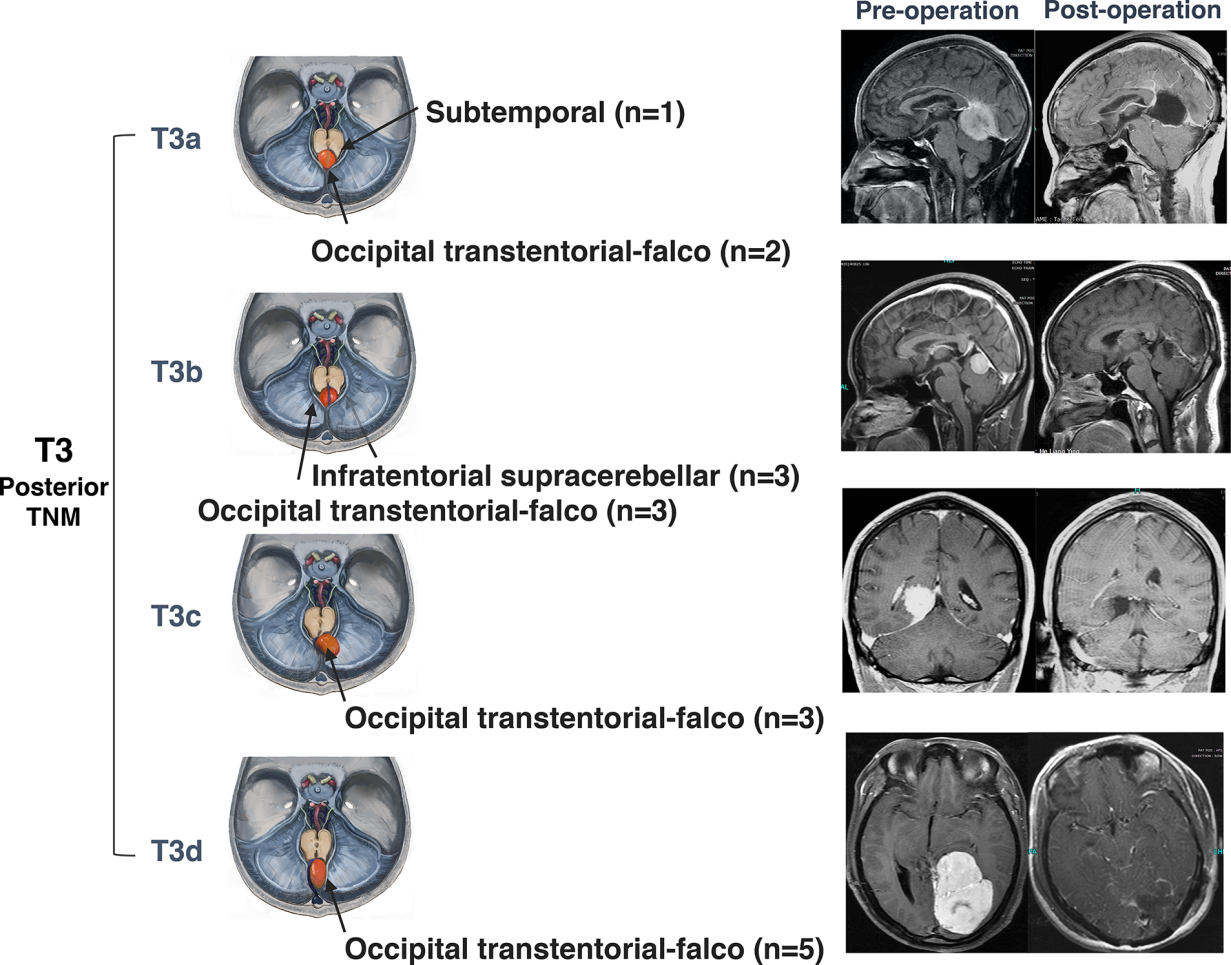
Surgical approaches and outcomes of posterior tentorial notch meningioma (TNM). Schematic diagram of surgical approaches and preoperative and postoperative MRI T1-weighted images with gadolinium-based contrast of further classification of posterior tentorial notch meningioma (T3). T3a = superior type, T3b = inferior type, T3c = lateral type, T3d= straight sinus type.

Surgeries for anterior and middle notch meningiomas were performed *via* the retrosigmoid approach (23), pterional approach (3), and pretemporal transcavernous approach (2). The subtemporal approach (6), combined subtemporal and retrosigmoid approach (1), and presigmoid sinus approach (1) were also applied (**Figure 5**). The surgical approaches for posterior notch meningiomas are dominated by the suboccipital transtentorial approach (13), infratentorial supracerebellar approach (4), and subtemporal approach (1) (**Figure 6**).

### Follow-up Methods

A combination of outpatient and telephone follow-ups was performed. The follow-up was initiated from the 3rd month after surgery, followed by the 6th and 12th month. If the patient was asymptomatic and the imaging data showed no tumor recurrence, the subsequent follow-up was elongated to once a year. If there is a possibility of tumor recurrence, it is recommended to increase the frequency of imaging examination. Those who experienced relapse or progression were suggested to undergo further treatment. All patients were followed-up routinely with MRI and physical examination. The end point of the follow-up of this study was June 1, 2019.

## Results

### Surgical Results

Among the 53 TNM patients, the maximum diameter of the tumors was 6.5 cm, the minimum was 2.0 cm, and the average diameter was 3.87 ± 0.97 cm, of which the anterior notch type’s average diameter was 3.87 ± 1.26 cm, the middle notch type was 3.64 ± 0.66 cm, and the posterior notch type was 4.18 ± 1.01 cm. No deaths occurred during the perioperative period and during follow-up. The surgical outcome was evaluated by pathological results, the relationship between the adjacent structures and tumor, and the degree of tumor resection. The outcomes of postoperative symptom improvement and complications are shown with the follow-up results (**Tables 1** and **2**).

**Table 1:** Neurological deficits after surgery.

Neurological deficits	Aggravation and new-onset										Total	Incidence
	*T1*			*T2*			*T3*					
	*T1a*	*T1b*	*T1c*	*T2a*	*T2b*	*T2c*	T3a	T3b	T3c	T3d		
Vision loss/Visual field defects	1	0	0	0	0	1	0	0	1	0	2	3.77%
Diplopia	4	0	1	0	1	2	0	0	0	0	8	15.10%
Trigeminal nerve dysfunction	1	0	0	0	1	2	0	0	0	0	4	7.55%
Hearing loss	1	2		2	0	0	0	0	0	0	5	9.43%
Facioplegia	0	1	0	1	0	0	0	0	0	0	2	3.77%
Limb weakness	0	0	2	0	0	1	0	0	0	0	3	5.66%

TNM, Tentorial notch meningioma.

**Table 2:** Postoperative improvements in neurological function.

Neurological deficits	Improvement/preoperative deficits												Total	Incidence
	*T1*				*T2*				*T3*					
	T1a	T1b	T1c		T2a	T2b	T2c		T3a	T3b	T3c	T3d		
Tinnitus and hearing loss	0/3				1/9		1/1		0/1	0/1			2/15	13.3%
Facioplegia/anterior hypogeusia		0/1			1/3		0/2	–	–				1/6	16.7%
Facial pain and numbness	1/4	2/2			1/3	2/2	1/3	–	–				7/14	50.0%
Unsteady gait and ataxia			3/3		5/7						0/1	0/2	8/13	61.5%
Limb weakness			1/3		0/1		0/3					2/3	3/10	30.0%
Vision loss/visual field defect	0/2			–	–						1/2	1/3	2/7	28.6%
Dysphagia			2/2		2/2			–	–				4/4	100.0%
Nausea and vomiting	–				1/1		1/1		1/1			1/2	4/5	80.0%
Consciousness disorder	1/1				–				1/1			1/1	3/3	100.0%
Diplopia	0/2				–				–				0/2	0%

TNM, Tentorial notch meningioma.

### Pathological Results

Postoperative paraffins were examined and the 53 TNMs were all benign meningiomas (WHO grade I), a total of one case of T1a, one case of T1b, and two cases of T3d showed regionally active cell hyperplasia. There were no atypical or malignant meningiomas in this group of cases.

### Composition of Adjacent Neurovascular Structure and Tumor

Preoperative symptoms, signs, imaging examination, and observation during surgery are included in the criteria for defining the neurovascular invasion of the tumor. The anterior notch type is complicated. CN III, IV, V, VI, and VII–VIII; were involved in different ways. Posterior circulation was mainly involved. The PCA, SCA, basilar artery (BA), and PComA were often displaced medially and posteriorly. The internal carotid artery (ICA) and middle cerebral artery (MCA) were displaced anteriorly and superiorly. The PCA was commonly enveloped by a tumor (five cases in this group). One case of severe invasion of the cavernous sinus and enveloping the internal carotid artery was observed.

The middle notch type mainly invaded CN V, VI, and VII–VIII. In individual cases, the P2 segment of the PCA and its branches were involved.

The posterior notch type was mainly correlated with the deep venous system such as Galen’s vein (Galen’s V), basilar veins, and internal cerebral veins (ICV). In addition, there were often branches of the PCA and SCA participating in the tumor’s blood supply. There was no involvement of CN IV in this series.

### Tumor Removal

According to the view of intraoperative observation and MRI within 72 h after surgery, the degree of surgical removal was divided into total removal, subtotal removal, and partial removal, and Simpson’s classification was referred at the mean time (14). Total removal corresponds to Simpson I and II, subtotal removal corresponds to Simpson III, which requires more than 90% of the original tumor volume being resected, and partial removal corresponds to Simpson IV or greater than 60% of the original tumor volume resected (**Table 3**).

**Table 3:** The tumor removal degree of the 53 TNM cases.

Type	Subtype	Total Removal	Subtotal Removal	Partial Removal
T1 (14)	T1a (5)	1	2	2
	T1b (6)	5	1	0
	T1c (3)	3	0	0
T2 (22)	T2a (17)	17	0	0
	T2b (2)	2	0	0
	T2c (3)	2	1	0
T3 (17)	T3a (3)	3	0	0
	T3b (6)	5	1	0
	T3c (3)	3	0	0
	T3d (5)	5	0	0
Total	53	46	5	2

TNM, Tentorial notch meningioma.

Regarding the 53 cases in our series, 46 cases of total removal (86.79%), 5 cases of subtotal removal (9.43%), and 2 cases of partial removal (3.77%) were achieved. Two of the partial resections were of the anterior notch type: a tumor remnant was left in the sella and posterior cranial fossa; the other case was extremely rich in blood supply and penetrated the arachnoid matter on the brainstem, which was too risky to achieve total removal.

One case of T2c TNM was resected through the retrosigmoid approach with a small supratentorial residual tumor left on the middle cranial fossa.

Seventeen posterior TNMs were totally resected, and three lateral subtype cases (T3a) achieved Simpson I. Six patients with Simpson II had venous sinus involvement. One patient underwent subtotal resection due to a huge calcification in the tumor, which needed to be removed by drilling, so the membrane structure closely adhering to the straight sinus was not forcibly removed.

### Follow-up

A total of 53 cases were studied in this group, of which 47 were effectively followed up, and 2 patients who were followed up for less than 3 months were not included in the follow-up results of tumor recurrence.

### Postoperative Neurofunction

Newly gained and aggravated postoperative neurological dysfunction include vision loss and visual field defects (2); diplopia (8); trigeminal nerve dysfunction (4) such as gum atrophy, chewing weakness, malocclusion, and facial numbness, which gradually appeared during follow-up; hearing loss and facioplegia (7); and limb weakness (3). The incidence of neurological dysfunction was analyzed in 53 cases (**Table 1**). Six cases were recorded by the condition at discharge due to loss to follow-up.

Neurological dysfunction that improved postoperatively include headache and dizziness (23), tinnitus and hearing loss (2), facioplegia and anterior hypogeusia (1), facial pain and numbness (7), unsteady gait and ataxia (8), limb weakness (3), vision loss and visual field defect (2), dysphagia (4), nausea and vomiting (4), and consciousness disorder (3). No diplopia improved (**Table 2**). One case was on a comatose state after two operations in another institution; two cases were patients with severe hydrocephalus.

### Postoperative Progression and Recurrence

A total of seven patients in this series did not meet the standard of total removal, five patients underwent gamma knife with stable disease, one patient without undergoing gamma knife suffered tumor progression during follow-up, and one patient lost to follow-up.

Regarding the anterior notch type (T1), a total of five cases did not meet the standard of total removal, and four cases followed medical prescription to undergo gamma knife showing no progress. One patient underwent gamma knife surgery until tumor recurrence, and the tumor was controlled afterwards. Overall, the recurrence rate of T1 TNM was 7.1%.

One case of subtotally removed mid-notch type (T2b) underwent gamma knife, and the tumor remnant was controlled. One case (T2a) had tumor recurrence 5 years after total removal, and no significant progress was observed after gamma knife treatment. The recurrence rate of T2 TNM was 4.5%.

One case of post-notch type recurred in the third year after surgery. Gamma knife was performed and resulted in stable disease (**Table 4**). The recurrence rate of T3 TNM is 5.9%.

**Table 4:** Tumor recurrence/progression and stereotactic radio therapy of the 53 TNM cases.

Type		Total removal (+)Recurrence (+)	Total removal (−)γ-knife (−)Progression (+)	Total removal (−)γ-knife (+)Progression (−)	γ-knife after recurrence/progression	Progression/Recurrence at the latest follow-up
	T1a	0	1	4	1	0
T1	T1b	0	0	0	0	0
	T1c	0	0	0	0	0
	T2a	1	0	0	1	0
T2	T2b	0	0	1	0	0
	T2c	0	0	0	0	0
T3	T3a	1	0	0	1	0
	T3b	0	0	0	0	0
	T3c	0	0	0	0	0
	T3d	1	0	0	1	0

TNM, Tentorial notch meningioma.

## Discussion

### Treatment for TNM

The majority of meningiomas are benign tumors, while the 53 cases of TNM in this series are all WHO I. For benign tumors, neurosurgeons should always pursue a thorough cure. However, because of the complexity of the tumor, it is generally accepted that for complex meningiomas, the goal of treatment is to prolong the period of stable disease and improve the quality of life as much as possible. Our principle is to achieve maximum removal of the tumor while maintaining a high quality of life.

The preferred treatment for meningioma is surgical resection. Stereotactic radiotherapy is an optional method for meningioma after surgical treatment. In 2015, Sheehan reported 675 cases of posterior cranial fossa meningioma treated with gamma knife during an average of 5 years of follow-up, with a control rate of 91.2% (15), but the average tumor diameter was approximately 2.08 cm, which is relatively small in this study. In our 53 cases, the maximum diameter of the tumors was 6.5 cm, the minimum was 2.0 cm, and the average diameter was 3.87 ± 0.97 cm; therefore, surgical resection was the first-line treatment for this clinical series. The patients who underwent subtotal resection were treated with gamma knife and achieved stable disease during follow-up, but the tumor residuals were also small (average diameter: 1.90 cm). For large tumor remnants, the long-term follow-up of stereotactic radiotherapy is needed for further exploration. Nevertheless, gamma knife is still an optional treatment for tumor remnants or inoperable small TNMs.

A series of 13 posterior TNMs reported by Bassiouni et al. achieved complete resection (Simpson grades 1 and 2) in 85% of patients. Permanent surgical morbidity was 23% (10). Samii et al. reported 25 TNM patients who underwent surgical resection with a radical surgical removal of 88% and no mortality in the follow-up data (16). A clinical study composed of 14 TNMs published in 2018 reported that complete tumor removal with resection/coagulation of dural attachment (Simpson I–II) was achieved in 9/14 patients (64.3%), while incomplete removal without coagulation of the dural attachment or with residual mass (Simpson III–IV) was achieved in 5/14 patients (35.7%) (17). Referring to the literature, the total resection rate and neurofunction preservation are still to be improved for neurosurgeons. We achieved 46 cases of total removal (86.79%), 5 cases of subtotal removal (9.43%), and 2 cases of partial removal (3.77%) in a total of 53 TNMs, with postoperative neurofunction data. Our surgical experience and approach proposal based on our detailed classification as well as the follow-up data of stereotactic radio therapy can be a reference for assisting future TNM treatment.

### Further Classification of TNM

The early classification of meningiomas is distinguished by their anatomic location. With the deepening of understanding of various types of meningiomas, additional types were further defined by the origin and extent of the tumor base, the relationship with important neurovascular structures, the location of the tumor body, and the choice of surgical approach. For example, anterior clinoidal meningioma (13) is divided into three types based on the tumor’s origin and its anatomic relationship with the ICA. With the gradually matured approach of neurosurgery, further classification allows for detailed treatment strategies, efficient management of potential complications, and improvement of the surgical outcome.

We believe that a reasonable surgical classification should meet the following criteria: First, preoperative examination or early intraoperative observation contributes to the classification being completed, which is feasible in clinical practice. Second, rational classification assists in selecting a suitable approach and predicting the prognosis. Third, a classification should conform to the objective distribution of the disease. To this end, based on the characteristics of this group of TNMs and our surgical experience, we further classified the three types of TNM based on the aforementioned TNM classification. Among them, T1b and T1c are often falsely diagnosed as petroclival meningioma on MRI, while T1a is easily misdiagnosed as parasellar meningiomas such as clinoid and cavernous sinus meningioma. However, the true origin of the tumor base of TNMs and these aforementioned tumors are different, which requires a totally different order of amputation of the tumor base for optimizing the blood supply management. In some cases, it is difficult to make a diagnosis relying on preoperative neuroimaging; therefore, intraoperative observation is required to confirm the origin of the tumor base for crafting the following surgical strategy.

### Surgical Approach Selection of TNM Based on Further Classification

Regarding anterior TNMs, the pterional and pretemporal transcavernous approach were applied for T1a (18–20). The reasons for such a choice are as follows: 1. The blood supply can be managed at an early stage. The blood supply to the tentorial notch often comes from the cerebellar marginal artery (21) originating from the meningohypophyseal trunk or directly from the cavernous segment of the ICA, which elongates superiorly on the roof of the cavernous sinus and posteriorly along the margin of the cerebellar tentorium to the straight sinus, supplying the CN III and IV, the wall of the cavernous sinus, inner 1/3 part of the cerebellum, and part of the falx related to the straight sinus (9). 2. The pretemporal transcavernous approach provides the possibility of managing tumors within the cavernous sinus. 3. The operating space of the pterional approach is limited at the margin of the cerebellum; however, for the T1a TNMs, the tumor body possesses a space-occupying effect. After internal debulking, the operating space is obviously increased, which compensates for the limitation of the exposure. 4. The oculomotor nerve and the trochlear nerve can be positioned early through a pretemporal transcavernous approach, and the free nerve mobility is increased, which facilitates neuroprotection intraoperatively. 5. The exposure of the cistern segment of the oculomotor nerve facilitates positioning of the arachnoid interface between the tumor and brainstem through both approaches (22).

The approach for T1b is dominated by the retrosigmoid approach. The tumor involving the Meckel’s cavity can be removed through the channel formed by breaking the petrosum or drilling the tuberculum of the internal acoustic canal (IAC) (16). In addition, TNMs without extending to the middle and inferior clivus and enveloping the basilar artery are always suitable for subtemporal transtentorial approach that facilitates early management of blood supply.

With regard to T1c, if the tumor does not involve the lower part of the mid-clivus, subtemporal transtentorial approach is suitable. In addition to the early transection of the blood supply, fully tentorium dissection can provide direct vision for managing the adhesion between the tumor and brainstem and posterior circulation, especially for the perforating vessels on the brainstem. If the lower part of the mid-clivus is involved, the retrosigmoid approach can be considered. The direction of the tumor displacing the brainstem, degree of compression, and adhesion should be noted. Operation should always be processed on the visualized interface without taking risk.

The mid-notch type is divided into the infratentorial type (T2a), supratentorial type (T2b), and supra-infratentorial type (T2c). Among the 22 cases of middle TNM, 17 cases were T2a, and the surgical approach was dominated by the retrosigmoid approach. This approach is easy to operate with satisfactory exposure to the cerebellopontine angle (CPA) and petroclival area, which is convenient for managing the involvement of the dorsal petrosum and has good exposure of the tentorium. These two T2b cases were totally resected through a subtemporal approach without manipulating the infratentorial structures. The T2c cases were separately processed using the presigmoid and combined subtemporal-retrosigmoid approach. The tumor base both involved the middle cranial fossa and the dorsal petrosum. The one with a larger extension underwent a presigmoid approach due to the wide visual field with which the tumor base could be managed from multiple perspectives with minimum blind point, while the disadvantage of this approach is the complicated procedure and the high risk of damage to the critical structure during petrosal drilling. The combined approach was chosen for the smaller T2b TNM to manage the tumor base posteriorly and anteriorly, avoiding damage to the temporal bone.

Regarding the 17 cases in our group of posterior TNM, except for one T3a case that underwent a subtemporal approach and 3 T3b cases that underwent infratentorial supracerebellar approach, the remaining 13 patients underwent the occipital transtentorial-falco approach. We believe that the occipital transtentorial-falco approach has the following characteristics: 1. A shorter operative distance; 2. Both occipital and longitudinal fissure areas can be utilized to achieve a wider lateral exposure, making it easier to separate the adhesion of the tumor and Galen’s system from multiple perspectives; 3. The infratentorial supracerebellar approach provides better exposure to the contralateral pineal region, which is more important for intra-axial tumors of the pineal region. However, for TNMs, the thick arachnoid of the quadrigeminal cistern provides a good interface to separate the tumor; 4. Dissection of the tentorium or falx allows removal of the tumor of the infratentorial region or contralateral falx (8); 5. There is no need to transect the drainage vein on the surface of the cerebellum; 6. Although the occipital lobe can be sagged laterally through gravity, stretching of the occipital lobe may cause visual field defects. It should be noted that during tentorium dissection, attention must be paid to the tentorial sinus, especially when the Galen’s vein or straight sinus is obstructed, since the tentorial sinus always compensates for drainage of the deep venous system (23).

For most TNMs, a single approach is capable of providing satisfactory exposure. However, for complex TNMs such as T1a and T2c, a single approach may be insufficient. The choice of surgical strategy is directly linked to prognosis. Choosing staging surgery, combined approach to remove the tumor, or more invasive and risky approach such as the presigmoid and subtemporal transpetrosal approach to remove the tumor is still challenging for neurosurgeons.

### Surgical Strategy

Situations inconsistent with the surgical plan may occur during the operation. For example, the hard tumor tightly integrates with the cavernous sinus, which leads to a diminished arachnoid interface, or even invasion of the adventitia of the blood vessel or tight adhesion between the tumor and brainstem. Rational judgment is required during unpredictable situations. Regarding brainstem adhesion, Samii believed that the surface of the tentorial meningioma is often covered with arachnoid, so there are multiple layers of membranes between the tumor and brainstem, which facilitates the safe dissection of the tumor (11). Nevertheless, the arachnoid interface may disappear in some cases with longer course and infiltrative growth, which greatly increases the difficulty of total removal and neurovascular protection. Many groups believe that leaving a thin residual tumor on the brainstem and blood vessels helps improve the quality of life. In one of our cases, preoperative MRI showed obvious brainstem edema, and there was still notable bleeding after cutting off the tumor blood supply. The brainstem surface participated in the tumor’s blood supply with tumor adhering to the BA, MCA, and SCA. Moreover, the arachnoid interface disappeared, so the tumor was not forcibly removed. A postoperative review of MRI showed that the residual tumor tissue on the surface of the brain stem was still enhanced. However, no obvious progression occurred during follow-up.

### Postoperative Neurodysfunction

Among the TNM patients in this series, dysfunction of eye movement is more common in the anterior and middle TNM, and related cranial nerves include CN III, IV, and VI, particularly T1a. These nerves may be heavily enveloped or deformed by the tumor. The increase in lateral force during the operation may increase nerve damage. The operation between the nerve intervals may cause disturbances in neurofunction. Finally, the region of nerves entering the dura mater and tumor base may share overlaps, and the blood supply of CN III and CN IV through the dura mater also originates from the tentorial notch artery branches (24, 25), which is consistent with the source of the TNM’s basal blood supply. Taken together, cranial nerves are easily damaged when managing the tumor base.

Portions of the middle TNMs often involve CN VII–VIII entering the IAC, and drilling the posterior wall of the IAC is necessary for total removal. CN VII–VIII is compressed by the tumor in the IAC, so piecemeal tumor resection may aggravate the nerve damage. The thermal or even physical effects during drilling of the IAC may directly damage CN VII–VIII. In addition, the arachnoid interface between the IAC and tumor may disappear, which makes it difficult to dissect the tumor safely. Meanwhile, the blood supply branches of CN VII–VIII from AICA often adhere to the tumor and even participate in the tumor blood supply. They are easily damaged during tumor debulking.

One case of anterior TNM with preoperative vision loss and postoperative aggravation showed CN II dysfunction. The other vision dysfunctional case is a posterior TNM with visual field defect due to occipital lobe stretching.

From our point of view, a reasonable surgical approach selection based on further classification is the foundation of total removal and neuroprotection for TNM. *In situ* decompression will relieve the lateral force to the nerves that have already been severely compressed by the tumor. Sharp separation with no penetration of the arachnoid interface is essential for separating nerve adhesions. Excessive disturbance of nerves and long-term traction should be avoided. Of note, tumor remnants on the cranial nerve are acceptable, and the surgical field can be continuously flushed by an assistant to reduce the thermal effect.

## Conclusion

Further TNM classification based on the intracranial location, extension direction, relationship with brainstem, and neurovascular structures guides preoperative evaluation, rational surgical approach selection, and surgical strategy formulation. The protection of the cranial nerve function of TNM is still difficult. It requires familiarity with the microanatomy of adjacent areas, skilled micromanipulation techniques, and rich experience. Taking microsurgery as the main body, a satisfactory outcome of TNM treatment can be achieved for complicated tumors by combining stereotactic radiotherapy.

## Data Availability Statement

The original contributions presented in the study are included in the article/supplementary materials. Further inquiries can be directed to the corresponding author.

## Ethics Statement

The studies involving human participants were reviewed and approved by the Ethics Committee of Xiangya Hospital. The patients/participants provided their written informed consent to participate in this study. Written informed consent was obtained from the individual(s) for the publication of any potentially identifiable images or data included in this article.

## Author Contributions

CQ, JW, WL, JY, XY, CZ, and QL performed the surgical procedures. CQ, JW, WL, YP, KX, CW, and JS performed data collection and analysis. CQ, JW, WL, and QL wrote the manuscript. QL supervised the entire study. All authors contributed to the article and approved the submitted version.

## Funding

This work was supported by grants from the National Key Technology Research and Development Program of the Ministry of Science and Technology of China (Grant number 2014BAI04B01) and the National Natural Science Foundation of China (Grant number 81802974). Grant 2014BAI04B01 had a role in the collection, analysis, and interpretation of data. Grant number 81802974 played a role in writing the manuscript.

## Conflict of Interest

The authors declare that the research was conducted in the absence of any commercial or financial relationships that could be construed as a potential conflict of interest.
